# Supplementary material for: Limitations of Recent Studies Dealing with the Antibacterial Properties of Silver Nanoparticles: Fact and Opinion
Source: Nanomaterials (Basel). 2019 Dec 13;9(12):1775. doi: 10.3390/nano9121775 (PMC6956306; doi:10.3390/nano9121775)
Supplement: Supplementary file 1 [file nanomaterials-09-01775-s001.pdf]

Table S1: Ag NPs and antibacterial activity evaluation of selected publications from 2015-2018 period.

Stabilizer	NPs size (nm)	Bacteria	Bacteria origin	Protocol	Culture media	CFU/mL	MIC (µg/mL)	MBC (µg/mL)	Disk loading	ZOI (mm)	Reference
"Naked"	19.5 ± 7.7	*E. faecalis*<br>*S. aureus*<br>*S. epidermidis*<br>*B. subtilis*<br>*E. coli*<br>*S. typhimurium*<br>*S. enterica*	KCCM 13807<br>KCTC 1916<br>KCTC 1971<br>KCTC 1021<br>KCTC 1682<br>KCCM 40253<br>KACC 10763	Kirby-Bauer method<br>Microdilution method<br>MIC90	Mueller Hinton Agar<br>Luria Bertani	10^7	256*<br>32–64*<br>32–64*<br>128*<br>128–256*<br>32–64*<br>256*	N/A	30 µg	20*<br>18*<br>21*<br>14*<br>16*<br>17*<br>16*	[3]
"Naked"	≈10	*A. baumannii* (*n* = 17)<br>*A. baumannii*<br>*A. nosocomialis* (*n* = 10)	Clinical isolates<br>ATCC 1906<br>Clinical isolates	Microdilution method	N/A	N/A	0.39–0.78<br>0.78<br>0.39–0.78	/	/	/	[4]
"Naked"	10<br>20<br>40	*E. coli*	N/A	Kirby-Bauer method	Mueller Hinton 2 Agar	N/A	/	/	N/A	1–2<br>1–2<br>1–2	[5]
"Naked"	55.6 ± 2.9 (DLS)	*P. aeruginosa* (*n* = 3)<br>*S. maltophilia* (*n* = 3)<br>*B. cepacian* (*n* = 3)<br>*S. aureus* (*n* = 3)	Clinical isolates	Kirby-Bauer method<br>Microdilution method<br>TKA	Cation Adjusted Mueller Hinton	1–5 × 10^9	1.06<br>1.06<br>4.25<br>2.125<br>2.125<br>1.06<br>4.25<br>4.25<br>4.25<br>8.5<br>8.5<br>8.5	2.125<br>2.125<br>4.25<br>4.25<br>4.25<br>2.125<br>4.25<br>4.25<br>4.25<br>17.0<br>8.5<br>8.5	N/A	N/A	[6]
"Naked"	2–5 (70–75% TEM)	*L. monocytogenes*	PCM 2191	Microdilution method	Tryptone Soy yeast extract broth	2 × 10^6	8	/	/	/	[7]
"Naked"	23.6 (TEM)<br>57.8 (DLS)	*E. coli* (K-12)	KCTC 1116	Growth Curves	Luria Bertani	1 × 10^8	/	/	/	/	[8]
"Naked"	10	*S. aureus*<br>*S. aureus* (*n* = 30)	ATCC 25923<br>Clinical Isolates	Agar Well diffusion method<br>Microdilution method	Mueller Hinton Agar<br>Luria-Bertani	2 × 10^8	12.5<br>12.5 (16)<br>25 (14)	25<br>12.5 (17)<br>25 (13)	N/A<br>12.5 µg/mL (18)<br>25 µg/mL (12)	25–30	[9]

N/A: not available; KCCM: Korean Culture Center of Microorganisms; KCTC: Korean Collection of Type Cultures; KACC: Korean Agriculture Culture Collection; MIC: minimal inhibitory concentration; ATCC: American Type Culture Collection; DLS: dynamic light scattering; TKA: Time Kill Assay; TEM: transmission electron microscope; PCM: Polish Collection of Microorganisms; *estimated.

Stabilizer	NPs size (nm)	Bacteria	Bacteria origin	Protocol	Culture media	CFU/mL	MIC (µg/mL)	MBC (µg/mL)	Disk loading	ZOI (mm)	Reference
“Naked”	40<br>(TEM)	*S. aureus*<br>*B. cereus*<br>*P. aeruginosa*<br>*K. pneumoniae*<br>*E. coli*	N/A	Agar Well diffusion method	Mueller Hinton Agar	N/A	/	/	N/A	27.7 ± 0.62<br>24.1 ± 0.80<br>15.8 ± 0.41<br>28.2 ± 1.07<br>29.4 ± 0.56	[10]
Unknown<br>“Naked”	35<br>27.2	*E. coli*<br>(K-12)	JM 109	Agar Well diffusion method	Mueller Hinton	1×10^4			N/A<br>20 µg/mL	5<br>7	[11]
Citrate	23 ± 2<br>(TEM)	*L. monocytogenes*<br>(*n* = 20)<br>*L. monocytogenes*	Clinical isolates<br><br>ATCC 13932	Colony Forming Units	Mueller Hinton	1×10^8	/	/	/	/	[12]
Citrate	6.0–28.2<br>(XRD)	*S. aureus*<br>*E. coli*	ATCC 25923<br>ATCC 35218	Kirby-Bauer method	Nutrient Agar	1×10^4	/	/	0.25–3<br>mmol/mL	10 to 25<br>3 to 11	[13]
Citrate	2.3 ± 0.5<br><br>12.5 ± 2.2<br><br>32.4 ± 6.5<br>(TEM)	*S. aureus*<br>*E. coli*<br><br>*S. aureus*<br>*E. coli*<br><br>*S. aureus*<br>*E. coli*	ATCC 35696<br>ATCC 23282<br><br>ATCC 35696<br>ATCC 23282<br><br>ATCC 35696<br>ATCC 23282	Microdilution method<br>Kirby-Bauer method<br>Growth Curves	Broth medium	10^8–10^9	7.8<br>7.8<br><br>15.6<br>15.6<br><br>62.5<br>62.5	15.6<br>15.6<br><br>31.3<br>31.3<br><br>125<br>125	62.5 µg/mL	18.8<br>19.5<br><br>14.8<br>16.2<br><br>9.3<br>11.0	[14]
Citrate	40–50<br><br>20 × 20–90	*S. aureus*<br>*B. subtilis*<br>*P. aeruginosa*<br>*K. pneumoniae*<br>*E. coli*<br><br>*S. aureus*<br>*B. subtilis*<br>*P. aeruginosa*<br>*K. pneumoniae*<br>*E. coli*	ATCC 25923<br>AST5-2<br>AL2-14B<br>AWD5<br>ATCC 25922<br><br>ATCC 25923<br>AST5-2<br>AL2-14B<br>AWD5<br>ATCC 25922	Microdilution method<br>Kirby-Bauer method<br>TKA	Nutrient Agar	10^8–10^9	190<br>195<br>188<br>184<br>190<br><br>358<br>350<br>348<br>320<br>340	N/A	242 µg	14*<br>15*<br>11*<br>17*<br>5*<br><br>10*<br>13*<br>9*<br>14*<br>6*	[15]
Citrate	20.1 ± 4.4<br>(TEM)	*E. coli*<br>*S. aureus*<br>*L. bulgaricus*<br>*L. casei*	ATCC 25922<br>ATCC 25923<br>CGMCC 1.6970<br>CGMCC 1.2435	TKA	Luria Bertani<br>Tryptone Soy<br>MRS	1×10^6	/	17.5<br>14<br>9<br>8	/	/	[16]
Citrate	10–40<br>(TEM)	*E. coli*	ATCC 25922	Growth Curves	Luria Bertani	/	/	/	/	/	[17]

N/A: not available; ATCC: American Type Culture Collection; DLS: dynamic light scattering; TKA: Time Kill Assay; TEM: transmission electron microscope; XRD: X-ray diffraction; CGMCC: China General Microbiological Culture Collection Center; MRS: deMan, Rogosa and Sharpe medium; *estimated from publication.

Stabilizer	NPs size (nm)	Bacteria	Bacteria origin	Protocol	Culture media	CFU/mL	MIC (μg/mL)	MBC (μg/mL)	Disk loading	ZOI (mm)	Reference
Citrate	20 ± 9<br>25 ± 3<br>11 ± 6<br>(TEM)	*S. aureus*	ATCC 25923	Agar Well diffusion method	Nutrient Agar	10^8–10^9	/	/	0.1 M ($AgNO_3$)	N/A	[18]
Citrate	42–58<br>(TEM)	*S. aureus*<br>*S. pyogenes*<br>*S. typhi*<br>*P. aeruginosa*	N/A	Kirby-Bauer method	Tryptone Soy	10^7	/	/	5μg/disc	14.8<br>13.6<br>12.5<br>19.1	[19]
Citrate	15	*E. coli*<br>*B. subtilis*	N/A	Kirby-Bauer method	Nutrient Agar	1.5 × 10^8	/	/	N/A	N/A<br>N/A	[20]
	18	*E. coli*<br>*B. subtilis*								7.0<br>6.5	
	30	*E. coli*<br>*B. subtilis*								7.5<br>7.5	
	30<br>(DLS)	*E. coli*<br>*B. subtilis*								N/A<br>N/A	
GSH	10–50	*C. jejuni*<br>(*n* = 22)	Animal or Human clinical isolates	Microdilution method	Brucella	1 × 10^8	9.85–39.4	9.85–39.4	/	/	[21]
		C. coli<br>(*n* = 18)					4.92–39.4	9.85–39.4			
		C. jejuni	NCTC 11168		Mueller Hinton		4.92	9.85			
D-xylose<br>L-arabinose<br>D-ribose<br>D-glucose<br>D-galactose<br>D-mannose<br>D-lactose<br>D-xylose	33<br>30<br>39<br>25<br>28<br>25<br>15<br>18	*E. coli*<br>*Klebsiella* spp.	N/A	N/A	N/A	N/A	N/A	N/A	N/A	N/A	[22]
Citrate	10.15 ± 3.37<br>(TEM)	*P. aeruginosa*<br>(PAO1)	N/A	Microdilution method	Luria Bertani without Chloride ions	1 × 10^8	17.5	75	/	/	[23]
L-fucose							7.5	40			
PEG	15.8 ± 2.2<br>(TEM)	*S. aureus*	ATCC 6538	Microdilution method	Mueller Hinton	N/A	4.7	/	/	/	[24]
		P. aeruginosa	ATCC 15442				2.3				
		S. enterica	ATCC 10708				2.3				
		E. coli	ATCC 11229				1.2				

N/A: not available; ATCC: American Type Culture Collection; DLS: dynamic light scattering; TEM: transmission electron microscope; GSH: glutathione; NCTC: National Collection of Type Culture; PEG: polyethylene glycol.

Stabilizer	NPs size (nm)	Bacteria	Bacteria origin	Protocol	Culture media	CFU/mL	MIC (µg/mL)	MBC (µg/mL)	Disk loading	ZOI (mm)	Reference
PC	3.3 ± 0.9<br>4.9 ± 2.9<br>(TEM)	*E. coli*<br>*S. aureus*	OW6<br>Mu50	Growth curves	Todd Hewitt broth	/	/	/	/	/	[25]
PVA	31	*E. coli*<br>*Pseudomonas sp*	N/A	Kirby-Bauer method	Nutrient Agar	N/A	/	/	N/A	3.4<br>4.1	[26]
	24	*E. coli*<br>*Pseudomonas sp*								4.9<br>6.2	
	19	*E. coli*<br>*Pseudomonas sp*								7.0<br>8.1	
	14<br>(SEM)	*E. coli*<br>*Pseudomonas sp*								8.8<br>10.2	
PVP/citrate	50–60<br>70–80	*E. coli*	N/A	Colony Forming Units	Nutrient Agar	/	/	/	/	/	[27]
PVP	14.0 ± 0.3<br>(TEM)	*E. coli*<br>(K-12)<br>*B. subtilis*	MG1655<br><br>ATCC 6051	Growth curves	Tryptone Soy	/	/	/	/	/	[28]
	5	*E. coli*<br>*P. aeruginosa*<br>*S. aureus*<br>*S. epidermidis*	ATCC 8739<br>ATCC 9027<br>ATCC 6538<br>ATCC 12228	Poisoned Food Technique	Mueller Hinton	1 × 10^8	1<br>2<br>2<br>4	/	/	/	[29]
	20	*E. coli*<br>*P. aeruginosa*<br>*S. aureus*<br>*S. epidermidis*	ATCC 8739<br>ATCC 9027<br>ATCC 6538<br>ATCC 12228				2<br>2<br>8<br>2				
	8	*A. hydrophila*<br>*P. putida*<br>*E. coli*<br>*B. subtilis*<br>*S. aureus*	4AK4<br>KT2442<br>Trans 1-T1<br>ATCC 28357<br>N/A	Kirby-Bauer method	N/A	3 × 10^8	No activity<br>No activity<br>No activity<br>No activity<br>10 µg/mL	/	10 µg/mL	0<br>0<br>0<br>0<br>15	[30]
	29<br>(TEM)	*A. hydrophila*<br>*P. putida*<br>*E. coli*<br>*B. subtilis*<br>*S. aureus*	4AK4<br>KT2442<br>Trans 1-T1<br>ATCC 28357<br>N/A	Microdilution method	Mueller Hinton		No activity<br>No activity<br>No activity<br>No activity<br>10 µg/mL			0<br>0<br>0<br>0<br>6	

N/A: not available; ATCC: American Type Culture Collection; DLS: dynamic light scattering; TKA: Time Kill Assay; TEM: transmission electron microscope; PCM: Polish Collection of Microorganisms; XRD: X-ray diffraction; CGMCC: China General Microbiological Culture Collection Center; GSH: glutathione; NCTC: National Collection of Type Culture; PEG: polyethylene glycol; CTAB: cetyl-trimethyl ammonium bromide; NTA: nanoparticle tracking analysis; PC: phosphorylcholine; PVA: polyvinyl alcohol; SEM: scanning electron microscope; PVP: polyvinylpyrrolidone.

Stabilizer	NPs size (nm)	Bacteria	Bacteria origin	Protocol	Culture media	CFU/mL	MIC (µg/mL)	MBC (µg/mL)	Disk loading	ZOI (mm)	Reference
PVP	15.6<br>(TEM)	*Citrobacter sp*<br>*Enterococcus sp*	N/A	Colony Forming Units	N/A	/	/	/	/	/	[31]
PVP	3–34<br>(TEM)	*S. aureus*<br>PTCC No. 1112<br>*E. coli*<br>PTCC No. 1330	ATCC 6537<br>ATCC 8739	N/A	Mueller Hinton	1×10^7	N/A	N/A	N/A	N/A	[32]
PVP	10–15<br>(TEM)	*E. coli*<br>*S. aureus*	ATCC 25922<br>ATCC 25923	Kirby-Bauer method	Nutrient Agar	1×10^7–10^8	/	/	100 µL	N/A	[33]
PVP	60 ± 15	*E. coli*	ATCC 25922	Growth curves<br>Microdilution method	Luria Bertani	10^3<br>10^4<br>10^5<br>10^6	50.0 ± 5.2<br>75.0 ± 2.6<br>100.0 ± 7.0<br>100.0 ± 6.0	/	/	/	[34]
PVP	55 ± 10	*E. coli*	ATCC 25922	Growth curves<br>Microdilution method	Luria Bertani	10^3<br>10^4<br>10^5<br>10^6	25 ± 4.6<br>37.5 ± 5.3<br>50.0 ± 3.5<br>75.0 ± 4.6	/	/	/	[34]
PVP	60 × 2000–4000	*E. coli*	ATCC 25922	Growth curves<br>Microdilution method	Luria Bertani	10^3<br>10^4<br>10^5<br>10^6	50.0 ± 4.6<br>100.0 ± 5.3<br>> 100.0<br>> 100.0	/	/	/	[34]
PVP	20.6 ± 3.1	*C. jejuni*<br>*(n = 4)*<br>*C. jejuni*<br>*Salmonella* spp.<br>*(n = 5)*	Chicken isolates<br>NCTC 11168<br>Chicken isolates	Microdilution method	Mueller Hinton<br>Luria Bertani	10^5	3.125–6.25<br>6.25<br>12.5–25	/	/	/	[35]
PVP/Glycerol	31.2<br>(TEM)<br>46.5<br>(DLS)	*C. sakazakii*	ATCC 29544<br>ATCC BAA894<br>ATCC 29004<br>ATCC 12868	Microdilution method<br>Oxford cup method	Luria Bertani	1×10^8	125<br>125<br>62.5<br>62.5	/	200 mg/L	14.3<br>13.8<br>16.3<br>16.0	[36]
Oleylamine	10<br>(TEM)	*B. subtilis*	ATCC 6633	Growth curves	Luria Bertani	/	/	/	/	/	[37]
Casein	12.5 ± 4<br>(TEM)<br>50.0 ± 0.7<br>(DLS)	*E. coli*<br>*P. aeruginosa*	MC 1061<br>DS 10-129	Bioluminescence inhibition assay	Luria Bertani	1×10^9	/	/	/	/	[38]

N/A: not available; ATCC: American Type Culture Collection; TEM: transmission electron microscope; NCTC: National Collection of Type Culture; PVP: polyvinylpyrrolidone.

Stabilizer	NPs size (nm)	Bacteria	Bacteria origin	Protocol	Culture media	CFU/mL	MIC (µg/mL)	MBC (µg/mL)	Disk loading	ZOI (mm)	Reference
Sericin	3.78 ± 1.14 (TEM)	*S. aureus*	ATCC 25923	Cell counting (FCM)	Nutrient medium	N/A	/	/	/	/	[39]
		E. coli	ATCC 25922								
Thioacetic acid	20–25	*S. aureus*	ATCC 25923	Microdilution method	Mueller Hinton	N/A	1	1	/	/	[40]
		S. epidermidis	ATCC 35984				1	10			
		A. baumannii	ATCC 19606				10	10			
		P. aeruginosa	ATCC 27853				10	10			
Propionic acid	30–35	*S. aureus*	ATCC 25923				1	1			
		S. epidermidis	ATCC 35984				1	1			
		A. baumannii	ATCC 19606				1	10			
		P. aeruginosa	ATCC 27853				10	10			
Lipoid acid	2.0 ± 0.5 (TEM)	*S. aureus*	N/A	Growth curves	Luria Bertani	1×10^6	/	/	/	/	[41]
		E. coli	DH5*α*								
		E. coli	DSM4230								
PEG	44	*E. coli*	N/A	Kirby-Bauer method	Nutrient Agar	N/A	/	/	6 µL	3	[42]
		Pseudomonas spp.								3	
EDTA	39	*E. coli*								4	
		Pseudomonas spp.								5	
PVP	35	*E. coli*								6	
		Pseudomonas spp.								6	
PVA	31 (SEM)	*E. coli*								7	
		Pseudomonas spp.								8	
"Naked" Unknown	7.5	*S. aureus*	ATCC 29737	Agar Well diffusion method	Brain Heart Infusion	1.5×10^8	9.7×10^{-8}	/		5–40*	[43]
		S. mutans	ATCC 35668				ND			5–22*	
		S. pyogenes	ATCC 8668				ND			8–20*	
		E. coli	ATCC 15224				ND			4–31*	
		P. vulgaris	ATCC 7829				ND			5–10*	
	10.1 (TEM)	*S. aureus*	ATCC 29737	Microdilution method	Mueller Hinton		4×10^{-9}			5–40*	
		S. mutans	ATCC 35668				4×10^{-8}			5–22*	
		S. pyogenes	ATCC 8668				4×10^{-8}			2–29*	
		E. coli	ATCC 15224				4×10^{-10}			6–39*	
		P. vulgaris	ATCC 7829				ND			3–5*	

N/A: not available; ATCC: American Type Culture Collection; DLS: dynamic light scattering; TEM: transmission electron microscope; PEG: polyethylene glycol; PVA: polyvinyl alcohol; SEM: scanning electron microscope; PVP: polyvinylpyrrolidone; EDTA: ethylenediaminetetraacetid acid; C12mim: 3-methylimidazolium chloride; FCM: Flow Cytometry; * estimated from publication.

Stabilizer	NPs size (nm)	Bacteria	Bacteria origin	Protocol	Culture media	CFU/mL	MIC (µg/mL)	MBC (µg/mL)	Disk loading	ZOI (mm)	Reference
PVA	10	*A. baumannii* (*n* = 17)<br>*P. aeruginosa* (*n* = 12)<br>*P. aeruginosa*<br>*P. aeruginosa*<br>*Enterobacteriaceae* (*n* = 21)<br>*S. maltophilia* (*n* = 2)<br>*S. aureus* (*n* = 13)<br>*S. aureus*<br>*S. epidermidis*<br>*Enterococcus sp* (*n* = 14)	Clinical isolates<br>Clinical isolates<br>ATCC 27853 S<br>INCQS 230<br>Clinical isolates<br>Clinical isolates<br>Clinical isolates<br>ATCC 29213<br>INCQS 198<br>Clinical isolates				MIC_{50}/MIC_{90}<br>13.5; ≥ 54<br>13.5; ≥ 54<br>13.5<br>≥ 54; ≥ 54<br>≥ 54; ≥ 54<br>≥ 54; ≥ 54<br>≥ 54<br>27<br>≥ 54; ≥ 54	≥ 54<br>≥ 54<br>27		0*	
Citrate	40	*A. baumannii* (*n* = 17)<br>*P. aeruginosa* (*n* = 12)<br>*P. aeruginosa*<br>*P. aeruginosa*<br>*Enterobacteriaceae* (*n* = 21)<br>*S. maltophilia* (*n* = 2)<br>*S. aureus* (*n* = 13)<br>*S. aureus*<br>*S. epidermidis*<br>*Enterococcus sp* (*n* = 14)	Clinical isolates<br>Clinical isolates<br>ATCC 27853 S<br>INCQS 230<br>Clinical isolates<br>Clinical isolates<br>Clinical isolates<br>ATCC 29213<br>INCQS 198<br>Clinical isolates	Agar Well diffusion method<br>Microdilution method<br>Time kill assay	Mueller Hinton<br>Mueller Hinton Cation Adjusted<br>Tryptone Soy<br>Mueller Hinton Cation Adjusted	N/A	3.4; 3.4<br>3.4; 3.4<br>3.4<br>6.7; 6.7<br>1.6; 1.6<br>6.7; 13.5<br>13.5<br>6.7<br>6.7; 13.5	6.7<br>27<br>6.7	50µL	12*	[44]
Citrate	60	*A. baumannii* (*n* = 17)<br>*P. aeruginosa* (*n* = 12)<br>*P. aeruginosa*<br>*P. aeruginosa*<br>*Enterobacteriaceae* (*n* = 21)<br>*S. maltophilia* (*n* = 2)<br>*S. aureus* (*n* = 13)<br>*S. aureus*<br>*S. epidermidis*<br>*Enterococcus sp* (*n* = 14)	Clinical isolates<br>Clinical isolates<br>ATCC 27853 S<br>INCQS 230<br>Clinical isolates<br>Clinical isolates<br>Clinical isolates<br>ATCC 29213<br>INCQS 198<br>Clinical isolates				≥ 10; ≥ 10<br>≥ 10; ≥ 10<br>≥ 10<br>≥ 10; ≥ 10<br>≥ 10; ≥ 10<br>≥ 10; ≥ 10<br>≥ 10<br>≥ 10<br>≥ 10; ≥ 10	≥ 10<br>≥ 10<br>≥ 10		N/A	

N/A: not available; ATCC: American Type Culture Collection; DLS: dynamic light scattering; TEM: transmission electron microscope; PEG: polyethylene glycol; PVA: polyvinyl alcohol; SEM: scanning electron microscope; * estimated from publication.

Stabilizer	NPs size (nm)	Bacteria	Bacteria origin	Protocol	Culture media	CFU/mL	MIC (μg/mL)	MBC (μg/mL)	Disk loading	ZOI (mm)	Reference
Cysteine	7.6 ± 1.5	*S. aureus*	ATCC 29213	Microdilution method	Mueller Hinton Cation Adjusted	N/A	580	/	/	/	[45]
		E. coli	ATCC 23716				145				
		P. aeruginosa	ATCC 25619				35				
PVP	7.7 ± 1.6 (TEM)	*S. aureus*	ATCC 29213				69				
		E. coli	ATCC 23716				69				
		P. aeruginosa	ATCC 25619				73				
Citrate	10.2 ± 2.3	*E. coli*	MG 1655	Growth curves	Luria Bertani	1×10^7	/	/	/	/	[46]
MPA	10.2 ± 2.5										
MHA	10.2 ± 2.2										
MPS	9.9 ± 2.0										
Citrate	40	*E. coli*	DH5α	Agar Well diffusion method Growth curves	Luria Bertani	N/A	/	/	4 x 10^{-9} mg	20.2 ± 5.5	[47]
PVP										0.8 ± 1.4	
PEG										24.5 ± 2.4	
Lipoid acid	9.5 ± 1.9 (TEM)	*Actinomyces* (*n* = 1)	Clinical isolates	Plate dilution method<br>Microdilution method	Brucella agar supplemented<br>Mueller Hinton	5×10^5	≤ 5	/	/	/	[48]
		Bacteroides (*n* = 4)	Clinical isolates				80 - ≥ 100				
		Bacteroides fragilis	ATCC 25285				≥ 100				
		Bifidobacterium (*n* = 1)	Clinical isolates				≤ 5				
		Bifidobacterium breve	ATCC 15700				10				
		Finegoldia (*n* = 2)	Clinical isolates				5-10				
		Fusobacterium (*n* = 4)	Clinical isolates				40 - ≥ 100				
		Fusobacterium nucleatum	ATCC 25585				40				
		Parabacteroides (*n* = 1)	Clinical isolates				80				
		Parvimonas (*n* = 2)	Clinical isolates				≤ 5				
		Peptostreptococcus (*n* = 1)	Clinical isolates				≤ 5				
		Peptostreptococcus anaerobius	ATCC 25286				10				
		Porphyromonas (*n* = 3)	Clinical isolates				≤ 5–20				
		Porphyromonas levii	ATCC 29147				≤ 5				
		Prevotella (*n* = 5)	Clinical isolates				10 - ≥ 100				
		Prevotella loescheii	ATCC 15930				≥ 100				
		Propionibacterium (*n* = 2)	Clinical isolates				10–40				
		Tannerella (*n* = 1)	Clinical isolates				20				
		S. aureus	ATCC 25923				5.0				
		S. aureus	ATCC 6538				2.5				
		S. aureus	ATTC 6538P				5.0				
		S. epidermidis	ATCC 14990				5.0				
		S. mutans	ATCC 29175				5.0				

N/A: not available; ATCC: American Type Culture Collection; TEM: transmission electron microscope; PEG: polyethylene glycol; PVP: polyvinylpyrrolidone; MPA: mercaptopropionic acid; MHA: mercaptohexanoic acid; MPS: mercaptopropionic sulfonic acid.

Stabilizer	NPs size (nm)	Bacteria	Bacteria origin	Protocol	Culture media	CFU/mL	MIC (µg/mL)	MBC (µg/mL)	Disk loading	ZOI (mm)	Reference
PEG	9.8 ± 2.0 (TEM)	*Actinomyces* (*n* = 1)	Clinical isolates	Plate dilution method<br>Microdilution method	Brucella agar supplemented<br>Mueller Hinton	5×10^5	≤ 5	/	/	/	[48]
		Bacteroides (*n* = 4)	Clinical isolates				40 - ≥ 100				
		Bacteroides fragilis	ATCC 25285				≤ 5				
		Bifidobacterium (*n* = 1)	Clinical isolates				≤ 5				
		Bifidobacterium breve	ATCC 15700				10				
		Finegoldia (*n* = 2)	Clinical isolates				5–20				
		Fusobacterium (*n* = 4)	Clinical isolates				40				
		Fusobacterium nucleatum	ATCC 25585				≤ 5				
		Parabacteroides (*n* = 1)	Clinical isolates				40				
		Parvimonas (*n* = 2)	Clinical isolates				≤ 5				
		Peptostreptococcus (*n* = 1)	Clinical isolates				≤ 5				
		Peptostreptococcus anaerobius	ATCC 25286				10				
		Porphyromonas (*n* = 3)	Clinical isolates				≤ 5–20				
		Porphyromonas levii	ATCC 29147				≤ 5				
		Prevotella (*n* = 5)	Clinical isolates				20 - ≥ 100				
		Prevotella loescheii	ATCC 15930				20				
		Propionibacterium (*n* = 2)	Clinical isolates				10–80				
		Tannerella (*n* = 1)	Clinical isolates				40				
		S. aureus	ATCC 25923				2.5				
		S. aureus	ATCC 6538				5.0				
		S. aureus	ATTC 6538P				10.0				
		S. epidermidis	ATCC 14990				0.625				
		S. mutans	ATCC 29175				10				

N/A: not available; ATCC: American Type Culture Collection; TEM: transmission electron microscope; PEG: polyethylene glycol.

Stabilizer	NPs size (nm)	Bacteria	Bacteria origin	Protocol	Culture media	CFU/mL	MIC (µg/mL)	MBC (µg/mL)	Disk loading	ZOI (mm)	Reference
"Naked"	11.2 ± 2.1 (TEM)	*Actinomyces* (*n* = 1)	Clinical isolates	Plate dilution method<br>Microdilution method	Brucella agar supplemented<br>Mueller Hinton	5×10^5	≤ 5	/	/	/	[48]
		Bacteroides (*n* = 4)	Clinical isolates				40 - ≥ 100				
		Bacteroides fragilis	ATCC 25285				≥ 100				
		Bifidobacterium (*n* = 1)	Clinical isolates				≤ 5				
		Bifidobacterium breve	ATCC 15700				≤ 5				
		Finegoldia (*n* = 2)	Clinical isolates				5–10				
		Fusobacterium (*n* = 4)	Clinical isolates				≤ 5–20				
		Fusobacterium nucleatum	ATCC 25585				≥ 100				
		Parabacteroides (*n* = 1)	Clinical isolates				≤ 5				
		Parvimonas (*n* = 2)	Clinical isolates				≤ 5				
		Peptostreptococcus (*n* = 1)	Clinical isolates				≤ 5				
		Peptostreptococcus anaerobius	ATCC 25286				≤ 5				
		Porphyromonas (*n* = 3)	Clinical isolates				≤ 5–10				
		Porphyromonas levii	ATCC 29147				≥ 100				
		Prevotella (*n* = 5)	Clinical isolates				≤ 5–40				
		Prevotella loescheii	ATCC 15930				≤ 5				
		Propionibacterium (*n* = 2)	Clinical isolates				≤ 5–10				
		Tannerella (*n* = 1)	Clinical isolates				10				
		S. aureus	ATCC 25923				2.5				
		S. aureus	ATCC 6538				2.5				
		S. aureus	ATTC 6538P				10.0				
		S. epidermidis	ATCC 14990				0.3125				
		S. mutans	ATCC 29175				10				
Citrate	15 ± 4	*E. coli* (K-12)	ATCC 10798	Microdilution method	Mueller Hinton	1×10^9	/	1.7×10^3	/	/	[49]
		E. coli	ER2566					165.0×10^3			
HH	13 ± 2	*E. coli* (K-12)	ATCC 10798					18.1×10^3			
		E. coli	ER2566					166.0×10^3			
SHSH	13 ± 4	*E. coli* (K-12)	ATCC 10798					5.2×10^3			
		E. coli	ER2566					10.3×10^3			
SHST	10 ± 6	*E. coli* (K-12)	ATCC 10798					2.6×10^3			
	(TEM)	*E. coli*	ER2566					55.0×10^3			
Starch	8 ± 4 (TEM)	*S. aureus*	N/A	Kirby-Bauer method	Nutrient agar	10^5	/	/	1×10^{-7} mol	11	[50]
		E. coli								9	
AOT	20	*E. coli*	N/A	Microdilution method	Luria Bertani	10^8	512	1024	/	/	[51]
		S. aureus					256	512			
	50	*E. coli*					1024	1024			
		S. aureus					512	2048			

N/A: not available; ATCC: American Type Culture Collection; TEM: transmission electron microscope; HH: Hydroxylamine hypochlorite; SHSH: Sodium hypophosphite and sodium hexametaphosphate; SHST: Sodium hypophosphite, sodium hexametaphosphate and sodium tripolyphosphate; AOT: Bis(2-ethylhexyl) sulfosuccinate.

References

3. Subbiah, R.; Jeon, S.B.; Park, K.; Ahn, S.J.; Yun, K. Investigation of cellular responses upon interaction with silver nanoparticles. *Int. J. Nanomedicine* **2015**, 191.
4. Łysakowska, M.E.; Ciebiada-Adamiec, A.; Klimek, L.; Sienkiewicz, M. The activity of silver nanoparticles (Axonnite) on clinical and environmental strains of Acinetobacter spp. *Burns* **2015**, *41*, 364–371.
5. Kourmouli, A.; Valenti, M.; van Rijn, E.; Beaumont, H.J.E.; Kalantzi, O.-I.; Schmidt-Ott, A.; Biskos, G. Can disc diffusion susceptibility tests assess the antimicrobial activity of engineered nanoparticles? *J. Nanoparticle Res.* **2018**, *20*, 62.
6. Pompilio, A.; Geminiani, C.; Bosco, D.; Rana, R.; Aceto, A.; Bucciarelli, T.; Scotti, L.; Di Bonaventura, G. Electrochemically Synthesized Silver Nanoparticles Are Active Against Planktonic and Biofilm Cells of Pseudomonas aeruginosa and Other Cystic Fibrosis-Associated Bacterial Pathogens. *Front. Microbiol.* **2018**, *9*, 1349.
7. Markowska, K.; Grudniak, A.M.; Milczarek, B.; Wolska, K.I. The Effect of Silver Nanoparticles on Listeria monocytogenes PCM2191 Peptidoglycan Metabolism and Cell Permeability. *Pol. J. Microbiol.* **2018**, *67*, 315–320.
8. Choi, Y.; Kim, H.-A.; Kim, K.-W.; Lee, B.-T. Comparative toxicity of silver nanoparticles and silver ions to Escherichia coli. *J. Environ. Sci.* **2018**, *66*, 50–60.
9. Manipriya, B.; Tasneem, B.; Prem, K.L.; Kalyani, M. Evaluation of antibacterial activity of silver nanoparticles against methicillin-resistant Staphylococcus aureus and detection of virulence factors-nuclease, phosphatase, and bio film production. *Asian J. Pharm. Clin. Res.* **2018**, *11*, 224.
10. Singh, V.; Tiwari, A. Evaluating the antimicrobal efficiency of chemically silver nanoparticles. *Int. J. Curr. Microbiol. Appl. Sci.* **2015**, *4*, 5–10.
11. Korshed, P.; Li, L.; Ngo, D.-T.; Wang, T. Effect of Storage Conditions on the Long-Term Stability of Bactericidal Effects for Laser Generated Silver Nanoparticles. *Nanomaterials* **2018**, *8*, 218.
12. Belluco, S.; Losasso, C.; Patuzzi, I.; Rigo, L.; Conficoni, D.; Gallocchio, F.; Cibin, V.; Catellani, P.; Segato, S.; Ricci, A. Silver As Antibacterial toward Listeria monocytogenes. *Front. Microbiol.* **2016**, *7*.
13. Skiba, M.; Pivovarov, A.; Makarova, A.; Vorobyova, V. Plasma-chemical Synthesis of Silver Nanoparticles in the Presence of Citrate. *Chem. J. Mold.* **2018**, *13*, 7–14.
14. Wu, Y.; Yang, Y.; Zhang, Z.; Wang, Z.; Zhao, Y.; Sun, L. A facile method to prepare size-tunable silver nanoparticles and its antibacterial mechanism. *Adv. Powder Technol.* **2018**, *29*, 407–415.
15. Acharya, D.; Singha, K.M.; Pandey, P.; Mohanta, B.; Rajkumari, J.; Singha, L.P. Shape dependent physical mutilation and lethal effects of silver nanoparticles on bacteria. *Sci. Rep.* **2018**, *8*, 201.
16. Tian, X.; Jiang, X.; Welch, C.; Croley, T.R.; Wong, T.-Y.; Chen, C.; Fan, S.; Chong, Y.; Li, R.; Ge, C.; et al. Bactericidal Effects of Silver Nanoparticles on Lactobacilli and the Underlying Mechanism. *ACS Appl. Mater. Interfaces* **2018**, *10*, 8443–8450.
17. Rojas-Andrade, M.; Cho, A.T.; Hu, P.; Lee, S.J.; Deming, C.P.; Sweeney, S.W.; Saltikov, C.; Chen, S. Enhanced antimicrobial activity with faceted silver nanostructures. *J. Mater. Sci.* **2015**, *50*, 2849–2858.
18. Vanitha, G.; Rajavel, K.; Boopathy, G.; Veeravazhuthi, V.; Neelamegam, P. Physiochemical charge stabilization of silver nanoparticles and its antibacterial applications. *Chem. Phys. Lett.* **2017**, *669*, 71–79.
19. Mostafa, A.A.; Sayed, S.R.M.; Solkamy, E.N.; Khan, M.; Shaik, M.R.; Al-Warthan, A.; Adil, S.F. Evaluation of Biological Activities of Chemically Synthesized Silver Nanoparticles. *J. Nanomater.* **2015**, *2015*, 1–7.
20. Khatoon, U.T.; Nageswara Rao, G.V.S.; Mohan, K.M.; Ramanaviciene, A.; Ramanavicius, A. Antibacterial and antifungal activity of silver nanospheres synthesized by tri-sodium citrate assisted chemical approach. *Vacuum* **2017**, *146*, 259–265.
21. Silvan, J.M.; Zorraquin-Peña, I.; Gonzalez de Llano, D.; Moreno-Arribas, M.V.; Martinez-Rodriguez, A.J. Antibacterial Activity of Glutathione-Stabilized Silver Nanoparticles Against Campylobacter Multidrug-Resistant Strains. *Front. Microbiol.* **2018**, *9*, 458.
22. Vasileva, M.Yu.; Ershov, A.Yu.; Baigildin, V.A.; Lagoda, I.V.; Kuleshova, L.Yu.; Shtro, A.A.; Zarubaev, V.V.; Yakimanskii, A.V. Synthesis of Silver Glyconanoparticles Based on 3-Thiopropionylhydrazones of Mono- and Disaccharides. *Russ. J. Gen. Chem.* **2018**, *88*, 109–113.
23. Bhargava, A.; Pareek, V.; Roy Choudhury, S.; Panwar, J.; Karmakar, S. Superior Bactericidal Efficacy of Fucose-Functionalized Silver Nanoparticles against *Pseudomonas aeruginosa* PAO1 and Prevention of Its Colonization on Urinary Catheters. *ACS Appl. Mater. Interfaces* **2018**, *10*, 29325–29337.
24. Nam, S.; Park, B.; Condon, B.D. Water-based binary polyol process for the controllable synthesis of silver nanoparticles inhibiting human and foodborne pathogenic bacteria. *RSC Adv.* **2018**, *8*, 21937–21947.
25. Sangsuwan, A.; Kawasaki, H.; Matsumura, Y.; Iwasaki, Y. Antimicrobial Silver Nanoclusters Bearing Biocompatible Phosphorylcholine-Based Zwitterionic Protection. *Bioconjug. Chem.* **2016**, *27*, 2527–2533.
26. Ajitha, B.; Ashok Kumar Reddy, Y.; Sreedhara Reddy, P. Enhanced antimicrobial activity of silver nanoparticles with controlled particle size by pH variation. *Powder Technol.* **2015**, *269*, 110–117.
27. Ashkarran, A.A. The effect of visible-light intensity on shape evolution and antibacterial properties of triangular silver nanostructures. *Opt. Mater.* **2016**, *58*, 454–460.
28. Garuglieri, E.; Cattò, C.; Villa, F.; Zanchi, R.; Cappitelli, F. Effects of sublethal concentrations of silver nanoparticles on *Escherichia coli* and *Bacillus subtilis* under aerobic and anaerobic conditions. *Biointerphases* **2016**, *11*, 04B308.
29. Li, W.-R.; Sun, T.-L.; Zhou, S.-L.; Ma, Y.-K.; Shi, Q.-S.; Xie, X.-B.; Huang, X.-M. A comparative analysis of antibacterial activity, dynamics, and effects of silver ions and silver nanoparticles against four bacterial strains. *Int. Biodeterior. Biodegrad.* **2017**, *123*, 304–310.
30. Mosselhy, D.A.; El-Aziz, M.A.; Hanna, M.; Ahmed, M.A.; Husien, M.M.; Feng, Q. Comparative synthesis and antimicrobial action of silver nanoparticles and silver nitrate. *J. Nanoparticle Res.* **2015**, *17*, 1–10.

31. Lau, C.P.; Abdul-Wahab, M.F.; Jaafar, J.; Chan, G.F.; Abdul Rashid, N.A. Toxic effect of high concentration of sonochemically synthesized polyvinylpyrrolidone-coated silver nanoparticles on Citrobacter sp. A1 and Enterococcus sp. C1. *J. Microbiol. Immunol. Infect.* **2017**, *50*, 427–434.
32. Asadi, M.; Khosravi-Darani, K.; Haj-Seyed Javadi, N.; Esmaeili, S.; Azadnia, E. Synthesis of Silver Nanoparticles through Chemical Reduction and its Antibacterial Effect. *RRJFPDT* **2015**, *3*, 18–23.
33. Gurusamy, V.; Krishnamoorthy, R.; Gopal, B.; Veeraravagan, V.; P, N. Systematic investigation on hydrazine hydrate assisted reduction of silver nanoparticles and its antibacterial properties. *Inorg. Nano-Met. Chem.* **2017**, *47*, 761–767.
34. Hong, X.; Wen, J.; Xiong, X.; Hu, Y. Shape effect on the antibacterial activity of silver nanoparticles synthesized via a microwave-assisted method. *Environ. Sci. Pollut. Res.* **2016**, *23*, 4489–4497.
35. Duffy, L.L.; Osmond-McLeod, M.J.; Judy, J.; King, T. Investigation into the antibacterial activity of silver, zinc oxide and copper oxide nanoparticles against poultry-relevant isolates of Salmonella and Campylobacter. *Food Control* **2018**, *92*, 293–300.
36. Wang, H.; Jiang, Y.; Zhang, Y.; Zhang, Z.; Yang, X.; Ali, Md.A.; Fox, E.M.; Gobius, K.S.; Man, C. Silver nanoparticles: A novel antibacterial agent for control of Cronobacter sakazakii. *J. Dairy Sci.* **2018**, *101*, 10775–10791.
37. Yi, J.; Cheng, J. Effects of water chemistry and surface contact on the toxicity of silver nanoparticles to Bacillus subtilis. *Ecotoxicology* **2017**, *26*, 639–647.
38. Bondarenko, O.M.; Sihtmäe, M.; Kuzmičiova, J.; Ragelienė, L.; Kahru, A.; Daugelavičius, R. Plasma membrane is the target of rapid antibacterial action of silver nanoparticles in Escherichia coli and Pseudomonas aeruginosa. *Int. J. Nanomedicine* **2018**, *Volume 13*, 6779–6790.
39. Lv, X.; Wang, H.; Su, A.; Chu, Y. A Novel Approach for Sericin-Conjugated Silver Nanoparticle Synthesis and Their Potential as Microbicide Candidates. *J. Microbiol. Biotechnol.* **2018**, *28*, 1367–1375.
40. Hoseini-Alfatemi, S.M.; Karimi, A.; Armin, S.; Fakharzadeh, S.; Fallah, F.; Kalanaky, S. Antibacterial and antibiofilm activity of nanochelating based silver nanoparticles against several nosocomial pathogens: Bioactivity of nanochelating based AgNPs. *Appl. Organomet. Chem.* **2018**, *32*, e4327.
41. Jin, J.-C.; Wu, X.-J.; Xu, J.; Wang, B.-B.; Jiang, F.-L.; Liu, Y. Ultrasmall silver nanoclusters: Highly efficient antibacterial activity and their mechanisms. *Biomater. Sci.* **2017**, *5*, 247–257.
42. Ajitha, B.; Kumar Reddy, Y.A.; Reddy, P.S.; Jeon, H.-J.; Ahn, C.W. Role of capping agents in controlling silver nanoparticles size, antibacterial activity and potential application as optical hydrogen peroxide sensor. *RSC Adv.* **2016**, *6*, 36171–36179.
43. Abbaszadegan, A.; Ghahramani, Y.; Gholami, A.; Hemmateenejad, B.; Dorostkar, S.; Nabavizadeh, M.; Sharghi, H. The Effect of Charge at the Surface of Silver Nanoparticles on Antimicrobial Activity against Gram-Positive and Gram-Negative Bacteria: A Preliminary Study. *J. Nanomater.* **2015**, *2015*, 1–8.
44. Cavassin, E.D.; de Figueiredo, L.F.P.; Otoch, J.P.; Seckler, M.M.; de Oliveira, R.A.; Franco, F.F.; Marangoni, V.S.; Zucolotto, V.; Levin, A.S.S.; Costa, S.F. Comparison of methods to detect the in vitro activity of silver nanoparticles (AgNP) against multidrug resistant bacteria. *J. Nanobiotechnology* **2015**, *13*, 64/1–64/16.
45. Padmos, J.D.; Boudreau, R.T.M.; Weaver, D.F.; Zhang, P. Impact of Protecting Ligands on Surface Structure and Antibacterial Activity of Silver Nanoparticles. *Langmuir* **2015**, *31*, 3745–3752.
46. Long, Y.-M.; Hu, L.-G.; Yan, X.-T.; Zhao, X.-C.; Zhou, Q.-F.; Cai, Y.; Jiang, G.-B. Surface ligand controls silver ion release of nanosilver and its antibacterial activity against Escherichia coli. *Int. J. Nanomedicine* **2017**, *Volume 12*, 3193–3206.
47. El-Zahry, M.R.; Mahmoud, A.; Refaat, I.H.; Mohamed, H.A.; Bohlmann, H.; Lendl, B. Antibacterial effect of various shapes of silver nanoparticles monitored by SERS. *Talanta* **2015**, *138*, 183–189.
48. Niska, K.; Knap, N.; Kędzia, A.; Jaskiewicz, M.; Kamysz, W.; Inkielewicz-Stepniak, I. Capping Agent-Dependent Toxicity and Antimicrobial Activity of Silver Nanoparticles: An *In Vitro* Study. Concerns about Potential Application in Dental Practice. *Int. J. Med. Sci.* **2016**, *13*, 772–782.
49. Kujda, M.; Ocwieja, M.; Adamczyk, Z.; Bochenska, O.; Bras, G.; Kozik, A.; Bielanska, E.; Barbasz, J. Charge stabilized silver nanoparticles applied as antibacterial agents. *J. Nanosci. Nanotechnol.* **2015**, *15*, 3574–3583.
50. Vu, X.H.; Duong, T.T.T.; Pham, T.T.H.; Trinh, D.K.; Nguyen, X.H.; Dang, V.-S. Synthesis and study of silver nanoparticles for antibacterial activity against *Escherichia coli* and *Staphylococcus aureus*. *Adv. Nat. Sci. Nanosci. Nanotechnol.* **2018**, *9*, 025019.
51. Feng, A.; Cao, J.; Wei, J.; Chang, F.; Yang, Y.; Xiao, Z. Facile Synthesis of Silver Nanoparticles with High Antibacterial Activity. *Materials* **2018**, *11*, 2498.
